# Burnout among nurses working in the primary health care centers in Saudi Arabia, a multicenter study

**DOI:** 10.3934/publichealth.2020065

**Published:** 2020-11-05

**Authors:** Mohammed Adeeb Shahin, Sami Abdo Radman Al-Dubai, Duoaa Seddiq Abdoh, Abdullah Saud Alahmadi, Ahmed Khalid Ali, Tamer Hifnawy

**Affiliations:** 1 Joint Program of Preventive Medicine Post Graduate Studies, Ministry of Health, Al-Madinah 41311, Saudi Arabia; 2 Medical Education Department, College of Dentistry, Taibah University, Al-Madinah 41311, Saudi Arabia; 3 Faculty of Medicine, Beni-Suef University, Egypt

**Keywords:** burnout, nursing, primary health care, Saudi Arabia, stress

## Abstract

Background: Burnout is a common psychosocial phenomenon among nursing. It has been attributed to prolonged exposure to stress in the work place. This study aimed to determine the prevalence and associated factors of burnout among nurses in the primary health care centers in Saudi Arabia. Methods: This cross-sectional study was conducted among 200 nurses by using a self-administered questionnaire. Maslach Burnout Inventory-Human Services Survey (MBI-HSS) was used to measure burnout. Results: Most participants were females (73.0%) and aged ≤35 years (52.0%). About 39% had high emotional exhaustion, 38% had high depersonalization and 85.5% had low personal accomplishment. About 89% (178) scored high at least on one subscale of burnout. Burnout was associated with age, educational level and sources of stress in the workplace. Conclusion: Level of burnout among nurses was high and was associated mainly with stressors in the workplace. Improving work environment and management of stress in the workplace should be a priority to minimize burnout among nurses.

## 1. Introduction

Burnout is a common psychosocial phenomenon among health care workers. World Health Organization (WHO) defined Burn-out as an occupational phenomenon, *“a syndrome conceptualized as resulting from chronic workplace stress that has not been successfully managed”* [1]. It is composed of three dimensions: emotional exhaustion (EE), characterized by the sensation of physical and mental overexertion and lack of energy; (ii) depersonalization (DP) characterized by emotional detachment and negative attitudes towards patients and colleagues; and (iii) low personal accomplishment (PA), the degree to which a person perceives doing well on worthwhile tasks [2]. Burnout had negative effects on the employees, by causing different physical and mental problems and also on the organization, by decreasing the quality of care provided for patients and decreasing productivity [3]. A recent meta-analysis study that investigated burnout among nursing found a prevalence of 28% for high emotional exhaustion, 15% for high depersonalization and 31% for low personal accomplishment [4]. Burnout was associated with many sources of stress in the workplace such as work overload, long working hours, lack of resources and conflict with colleagues in addition to sociodemographic characteristics such as gender, age and years of experience. Previous studies in Saudi Arabia have been conducted among nurses in tertiary hospitals but not in the primary health care centers [5]–[8]. This study aimed to determine the prevalence and associated factors of burnout among nurses working in the primary health care centers in Medina city, Saudi Arabia.

## 2. Materials and methods

### 2.1. Study setting and sample

This observational analytical cross-sectional study was conducted among 200 nurses in the primary health care centers (PHC) in Medina city, Saudi Arabia. Al Madinah was divided into four regions, and three PHC centers were selected randomly from each region. All nurses in each center were approached. Those who had an experience of less than one-year were excluded.

### 2.2. Study instruments

A self-administered questionnaire consisting of three parts was used in this study. The first part included questions on the sociodemographic characteristics. Level of education was categorized into two categories; Bachelor of Science Nursing (BSN; 4 years study and one-year internship) and Diploma in nursing (DN: three years study and 6 months internship).

The second part assessed burnout by using the validated Maslach Burnout Inventory-Human Services Survey (MBI-HSS) which is the most commonly used tool for assessing burnout. It consists of 22 items which are divided into three subscales: emotional exhaustion, 9 items (the feelings of being emotionally overrun and exhausted by one's work); depersonalization, 5 items (the tendency to view others as objects rather than as feeling persons) and personal accomplishment, 8 items (the degree to which a person perceives doing well on worthwhile tasks). The items are answered in a 7-point scale ranging from 0 (never) to 6 (every day) [2]. The three scores are calculated for each respondent. High scores for EE and DP indicated higher levels of burnout, while high scores for PA indicated lower levels of burnout. This instrument was validated in many languages including Arabic language [9]. Cronbach's alpha coefficient for the three MBI subscales of the Arabic version were: emotional exhaustion (alpha = 0.88), depersonalization (alpha = 0.78), personal accomplishment (alpha = 0.89) [9] High level of burnout is defined in this study as high score on any of the three subscales of burnout [9],[10]. Sources of stress were assessed by 10 items which were obtained from the literature [10]. These items were headed by the following question: *“to which extent dose the following conditions cause stress to you”*. Each item was scored from zero (causing no stress) to 4 (causing severe stress) [10].

### 2.3. Ethical issues

Ethical approval was obtained from the Ethics Committee of the Directorate of Health in Al-Madinah. Objectives and benefits of the study were explained to the participants. Participants confidentiality and anonymity were assured. Signed consents were obtained from the participants.

### 2.4. Data analysis

Analysis was performed using Statistical Package for the Social Sciences (SPSS®) (version 22.0, IBM, Armonk, NY). The 22 items of MBI were summed to obtain the total score of each subscale [2].

Each subscale was categorized into low, moderate and high according to the recommended cut-off points [9]. Test of normality was performed for each subscale. T-test and analysis of variance (ANOVA) test were used to assess the association between burnout subscales and the sociodemographic variables. Pearson Correlation coefficient was used to assess the association between burnout subscales and the sources of stress. To obtain the significant factors associated with each subscale of burnout, multiple linear regression analysis was employed by using “Backward” technique. Multi-collinearity was checked between the independent variables by using the VIF. The accepted level of significance was below 0.05 (p < 0.05).

## 3. Results

### 3.1. Socio-demographics of the participants

Most participants were females (73.0%), aged ≤35 years (52.0%), married (81.0%) and had >10 years of service. Most of them had no administrative work (80.0%), had diploma (75.0%) and had a monthly income of less than 12 thousand Saudi Rial (SAR) (53.0%) (Table 1).

**Table 1.** Socio-demographic characteristics of the participants.

	n	%
Age		
≤35	104	52.0
>35	96	48.0
Gender		
Male	54	27.0
Female	146	73.0
Marital status		
Single	30	15.0
Married	162	81.0
Divorced/widower	8	4.0
Educational level		
Diploma	150	75.0
Bachelor	50	25.0
Years of service		
5 or less	32	16.0
6–10	57	28.5
>10	111	55.5
Administrative task		
Yes	40	20.0
No	160	80.0
Monthly income (SAR)*		
<12000	106	53.0
≥12000	94	47.0

Note: *1 USD = 3.7 SAR.

### 3.2. Prevalence of burnout and sources of stress

About 39% had high EE, 38% had high DP and 85.5% had low PA. Forty-five participants (22.5%) scored high on all the three subscale of burnout and 178 scored high at least on one subscale of burnout (89%) (Table 2). The reliability analysis of the three subscales yielded Cronbach alpha of 0.84 for EE, 0.76 for DP and 0.85 for PA.

**Table 2.** Prevalence of burnout among participants.

	Low n (%)	Moderate n (%)	High n (%)
EE	72 (36)	50 (25)	78 (39)
DP	46 (23)	78 (39)	76 (38)
PA	171 (85.5)	11 (5.5)	18 (9.0)

The most important sources of stress were long working hours, work overload, fear of violence and lack of resources (Table 3).

**Table 3.** Sources of stress in the workplace ranked by mean.

Item	Mean
Long working hours	3.161
Work overload	2.779
Fear of violence	2.623
Lack of resources	2.588
Work demands affect my personal homelife	2.362
Fear of making mistake that can lead to serious consequences	2.302
Working with uncooperative colleagues	2.302
Poor work environment	2.281
Office work	1.985
Cannot participate in decision-making	1.995

### 3.3. Factors associated with burnout in univariate analysis

**Table 4.** Relationship between burnout and socio-demographic characteristics.

Variables	Nurses' burnout					
	EE		DP		PA	
	Mean (SD)	P value	Mean (SD)	P value	Mean (SD)	P value
Age						
≤35	21.4 (11.5)		8.9 (5.0)		21.2(5.8)	
>35	24.9 (13.5)	0.041	9.6 (6.4)	0.348	16.1(5.5)	0.002
Gender						
Male	23.4 (13.4)		9.2 (5.2)		17.9 (6.2)	
Female	22.9 (12.2)	0.807	9.3 (5.1)	0.855	19.0 (5.0)	0.502
Marital status						
Single	22.2 (13.1)		8.9 (5.2)		20.0(5.4)	
Married	23.2 (12.5)		9.3 (5.7)		18.3(5.1)	
Divorced/widower	22.6 (13.8)	0.910	9.3 (7.2)	0.926	17.7(6.2)	0.794
Educational level						
Diploma	21.2 (12.8)		9.2 (5.8)		20.8 (5.4)	
University	28.4 (10.3)	<0.001	9.4 (5.5)	0.800	14.2 (7.2)	0.001
Years of service						
5 or less	22.8 (12.1)		8.3 (4.5)		22.8 (4.2)	
6–10	21.9 (11.3)		9.4 (5.3)		19.4 (6.4)	
>10	23.7 (13.3)	0.658	9.4 (6.2)	0.627	17.3 (5.7)	0.060
Administrative task						
Yes	22.4 (11.2)		8.7 (5.4)		23.2 (6.5)	
No	23.2 (12.9)	0.727	9.4 (5.8)	0.464	17.7 (7.4)	0.021
Monthly income (SAR)						
<12000	24.6 (11.8)		10.5 (5.5)		26.3 (6.4)	
≥12000	22.0 (12.9)	0.138	8.4 (5.7)	0.014	25.5 (5.6)	0.921

In univariate analysis, emotional exhaustion score was significantly higher among those aged >35 years (24.9 ± 13.5) compared to those aged ≤35 years (21.4 ± 11.5), (p = 0.041), and among those who had Bachelor degree (28.4 ± 10.3) compared to those who had diploma (21.2 ± 12.8), (p < 0.001) (Table 4).

EE was correlated positively and significantly with all the ten sources of stress (r coefficient ranged from 0.379 to 0.586), (p < 0.001) (Table 5). DP was higher among those who had an income of <12000 SAR (10.5 ± 5.5) compared to those with income of ≥12000 (8.4 ± 3.7), (p = 0.014) (Table 4). DP was correlated positively and significantly with all the ten sources of stress (r coefficient ranged from 0.198 to 0.368), (p < 0.005) (Table 5). PA was significantly lower among those who aged >35 years (16.1 ± 5.5) compared to those aged ≤35 years (21.2 ± 5.8), (p = 0.002), among those who had university degree (14.2 ± 7.2) compared to those who had diploma (20.8 ± 5.4), (p = 0.001) and among those who had not administrative task (17.7 ± 7.4) compared to those who had (23.2 ± 6.5), (p = 0.021) (Table 4).

**Table 5.** Relationship between burnout and sources of stress in the workplace.

Item	EE		DP		PA	
	Coefficient	P value	Coefficient	P value	Coefficient	P value
Work overload	0.495	<0.001	0.204	0.004	−0.106	0.135
Long working hours	0.379	<0.001	0.198	0.005	−0.007.	0.926
Fear of violence	0.422	<0.001	0.216	0.002	−0.100	0.161
Poor work environment	0.586	<0.001	0.368	<0.001	−0.219	0.002
Lack of resources	0.511	<0.001	0.301	<0.001	−0.086	0.228
Fear of making mistake that can lead to serious consequences	0.428	<0.001	0.220	<0.001	−0.097	0.174
Working with uncooperative colleagues	0.362	<0.001	0.197	0.005	−0.064	0.365
Office work	0.340	<0.001	0.273	<0.001	−0.055	0.436
Cannot participate in decision-making	0.424	<0.001	0.329	<0.001	−0.028	0.697
Work demands affect my personal home life	0.525	<0.001	0.249	<0.001	−0.0091	0.200

### 3.4. Factors associated with burnout in multivariate analysis

In multivariate analysis, significant predictors of EE were work overload (p = 0.010), poor work environment (p < 0.001), lack of resources (p = 0.033), working with uncooperative colleagues (p = 0.005), work demands affect personal homelife (p < 0.001) and having university education (p < 0.001) (Table 6). Significant predictors of DP were poor work environment (p < 0.001), “cannot participate in decision-making” (p = 0.041) and low income (<12000 SAR) (Table 6). Low personal accomplishment was significantly predicted by age (>35 years) (p=0.001), educational level (university), (p = 0.004) and no administrative task (p = 0.003) (Table 6).

**Table 6.** Factors associated with burnout in multivariate analysis.

	B	SE	Beta	P value	VIF
Emotional exhaustion					
Work overload	1.636	0.63	0.170	0.010	1.688
Poor work environment	3.134	0.68	0.318	<0.001	1.887
Lack of resources	1.473	0.68	0.148	0.033	1.853
Fear of making mistake that can lead to serious consequences	1.038	0.59	0.110	0.082	1.565
Working with uncooperative colleagues	1.875	0.65	−0.199	0.005	1.907
Work demands affect my personal home life	2.226	0.56	0.267	<0.001	1.774
University (reference = diploma)	6.009	1.51	0.206	<0.001	1.052
Depersonalization					
Poor work environment	1.246	0.35	0.278	<0.001	1.246
Cannot participate in decision-making	0.702	0.35	0.156	0.041	0.702
Monthly income less than 12K SAR	−1.776	0.76	−0.0152	0.021	−1.776
Personal accomplishment					
Age (>35)	−5.550	1.59	−0.0234	0.001	1.009
Educational level (Bachelor)	−5.354	1.82	−0.0196	0.004	1.001
No administrative task	−5.960	1.98	−0.0201	0.003	1.010

## 4. Discussion

The primary aim of this study was to estimate the prevalence of burnout and its associated factors among nurses in the primary health care setting. This study found 89% of the participants scored high at least on one subscale of burnout. Low personal accomplishment was found among 89% of nurses while high EE and high DP were reported by 39%, and 38% respectively. Moderate level of burnout was found among 25% (EE), 39% (DP) and 5.5% (PA). The overall prevalence of burnout in this study was 89%.

Previous studies among nurses in Saudi Arabia found that 32 % to 71.6% of nurses had high levels of burnout [5]–[8]. It was found by Al-Turki et al. that 45% of nurses had high EE, 42% had high depersonalization and 71.5% had low personal accomplishment. [5] Another study from Saudi Arabia found that 71.6 % of nurses had high level of burnout. [7] Another study from Saudi Arabia reported that 42% of nurses had moderate level of stress. [8] However, these two previous studies did not define the cut-off point for burnout. All the other mentioned studies used Maslach burnout inventory.

A recent study from Egypt found that 54.6% of nurses had average levels of emotional exhaustion, 48% scored high on depersonalization, and 77.5% had low personal accomplishment [11]. Another study from Egypt found that 52.8% of nurses experienced high EE, 7.2% had high level of DP and 96.5% had low PA [12]. A study among Iranian nurses found that 25% of the participants had high level of burnout. [13] A study of nurses in Israel reported that 30.8% reported high emotional exhaustion, 5.1% had high depersonalization, and 84.6% had low personal accomplishment [14]. In Jordan 55% of nurses reported high level of emotional exhaustion, 50% reported high level of depersonalization, and 50% reported low personal accomplishment [15]. A recent international meta-analysis study that investigated burnout among nurses found that 28% of nurses had high level emotional exhaustion, 15% had high level of depersonalization and 31% had low personal accomplishment [4]. Regarding factors associated with burnout, this study found that high emotional exhaustion was associated with age group, level of education, and with sources of stress in the work place such as work overload, lack of resources, uncooperative colleagues, and poor working environment. DP was associated with low income, poor working environment and inability to participate in decision-making. Low PA was associated with age group, level of education and no engagement in administrative work.

While some studies had not found association between burnout and socio-demographic factors, [8] some other studies had found a significant association between burnout and age, marital status and education level [5],[7]. However, there is a great agreement between studies that burnout is associated with stress and sources of stress in the workplace [15]–[20].

That sources of stress in the workplace included role conflict, work overload, conflict with colleagues, long working hours, poor working environment and low supervisor support. A previous meta-analysis study found that job insecurity, low job control, low reward, high demands and high work load increased the risk for developing burnout [21].

Long-term exposure to stressors was found to affect the professional quality of life, leading to cognitive and emotional distress and burnout [22]. Continuous effort in stressful, demanding tasks can have physiological and psychological impacts, such as increased heart rate and prolonged stimulation of the sympathetic nervous system. This is well recognized to be associated with exhaustion, particularly when the workload is high. Long working hours was found to be associated with emotional exhaustion because it produces excessive demands and disrupt family life and ability to trail outside interests [15]–[17].

This finding emphasis that any effort to manage burnout should be directed toward the management of sources of stress in the work place. Burnout was also found to be affected by other factors rather than work related factors and stressors in the work place. It was found in the previous studies that Alexithymic personality trait increased likelihood to experience burnout and has a negative effect on the professional quality of life among. radiation oncologists [23],[24]. In addition, emotional intelligence was found to be linked with all the three parts of burnout [25]. Emotional intelligence is defined as the ability to perceive emotion, integrate emotion to facilitate thought, understand emotions, and regulate emotions to promote personal growth [26].

## 5. Conclusions and future directions

This study found that 89% of the participants scored high at least on one subscale of burnout. Burnout was associated mainly with work related sources of stress. A comprehensive interventional approach is needed to minimize and prevent burnout among nurses in the primary health care centers. There were three types of interventions to manage burnout: individual-focused, organizational, and combine interventions. Individual-focused interventions included self-care workshops, stress management skills, communication skills training, yoga, mindfulness, meditation and coping programs. Organizational interventions aimed mainly to reduces stress and to mitigate the impact of stressors in the workplace; they included workload or schedule-rotation, stress management training program, access to peer mentoring, help and guidance from experienced work colleagues and teamwork/transitions. Individual and organizational interventions should be combined to effectively reduce burnout among healthcare providers. It would be also of great interest if future studies investigate which personality factors are associated with burnout in nursing working in primary health care centers. This will help to prioritize intervention to focus on nurses with high risk personality trait. Interventions to improve emotional intelligence are also recommended. Poulsen & Poulsen (2018) proposed a Self-Determination Theory and they suggested two steps to prevent burnout during early career. The first step was to educate trainers and trainees about times when individuals may be vulnerable to work stress. Learning how to recognize the warning signs of burnout and being aware of vulnerability is a vital first step. Education about the need for self-awareness and importance of self-care would occur in the early stages of training. The next step involved alerting practitioners regarding the extent and accessibility of information regarding evidence-based strategies that can be employed to address exhaustion and prevent disengagement [27].
